# The potential benefits of monitoring oxygen delivery in relation to O_2_ERi and VCO_2_ during normothermic regional perfusion in DCD donors

**DOI:** 10.1051/ject/2024028

**Published:** 2024-12-20

**Authors:** Ignazio Condello

**Affiliations:** 1 Department of Cardiac Surgery, Anthea Hospital Via Camillo Rosalba, n 35 7014 Bari Italy

As the field of organ transplantation continues to evolve, the use of Donation after Circulatory Death (DCD) donors has emerged as a critical method to expand the donor pool. However, the preservation of organs from DCD donors remains a significant challenge, primarily due to the risk of ischemic damage and subsequent organ dysfunction. Normothermic Regional Perfusion (NRP) has been introduced as a technique to mitigate these risks, restoring circulation and oxygenation in the donor organs. Within this context, the monitoring of indexed oxygen delivery (DO_2*i*_) about Equilibrium between Required and Supplied Oxygen (O_2_ERi) and carbon dioxide production (VCO_2_) offers substantial potential benefits that can significantly enhance the outcomes of organ transplantation. Indexed Oxygen delivery (DO_2*i*_) plays a pivotal role in ensuring the viability of organs during NRP. Adequate DO_2*i*_ is essential to maintain cellular metabolism and prevent ischemic injury during the period between circulatory cessation and organ retrieval. The study by de Somer et al. (2011) highlights the critical nature of maintaining optimal DO_2*i*_ levels during cardiopulmonary bypass, demonstrating that inadequate DO_2*i*_ is strongly associated with the development of acute kidney injury (AKI) [1]. This finding underscores the importance of monitoring and maintaining adequate DO_2*i*_ levels not only during cardiopulmonary bypass but also in the context of NRP for DCD donors, where organ preservation is paramount. The Equilibrium between Required and Supplied Oxygen (O_2_ERi) is a crucial metric that reflects the balance between the oxygen provided to the tissues and the oxygen they actually consume. Maintaining this balance is vital to prevent both under-oxygenation, which can lead to ischemic injury, and over-oxygenation, which can cause oxidative stress and tissue damage. The work by Ranucci et al. (2024) introduced the concept of a multifactorial dynamic perfusion index that incorporates DO_2*i*_ and other perfusion-related variables, demonstrating improved predictive accuracy for AKI when these factors are considered together. In the context of NRP, monitoring O_2_ERi in relation to DO_2*i*_ could enable more precise control over the oxygenation status of the organs, helping to optimize their viability for transplantation [2]. A study my group and I published in 2020 showed that hyperlactatemia during CPB, often a sign of inadequate oxygen delivery relative to metabolic demands, can be predicted by monitoring DO_2*i*_ in relation to O_2_ERi [3].

O_2_ERi is a metric used to assess the balance between the oxygen delivered to tissues (DO_2*i*_) and the oxygen they consume. It is essentially the oxygen extraction ratio indexed to a patient’s body surface area (BSA). This balance is critical to prevent both under-oxygenation (leading to ischemia) and over-oxygenation (which can cause oxidative stress).

O_2_ERi can be calculated using the following formula:

$$O_{2}\mathrm{ERi}={\mathrm{VO}}_{2i}/{\mathrm{DO}}_{2i}.$$

Where:

VO_2*i*_: The indexed volume of oxygen consumed by the tissues per minute, indexed to BSA.

DO_2*i*_: The indexed oxygen delivery, which is the amount of oxygen delivered per minute, indexed to BSA.

VO_2*i*_ is generally calculated as:

$${\mathrm{VO}}_{2i}=\left(\mathrm{Arterial}\;\mathrm{Oxygen}\;\mathrm{Content}-\mathrm{Venous}\;\mathrm{Oxygen}\;\mathrm{Content}\right)\times \mathrm{Blood}\;\mathrm{Flow}\times 10/\mathrm{Body}\;\mathrm{Surface}\;\mathrm{Area}.$$

DO_2*i*_ is generally calculated as:

$${\mathrm{DO}}_{2i}=\mathrm{Blood}\;\mathrm{Flow}\;\times (\mathrm{Arterial}\;\mathrm{Oxygen}\;\mathrm{Content}/\mathrm{Body}\;\mathrm{Surface}\;\mathrm{Area})$$

[3]

In clinical settings, VO_2_ is often approximated using indirect calorimetry or can be calculated if CO_2_ production (VCO_2_) and respiratory quotients (RQ) are known. The relationship between DO_2*i*_ and VCO_2_ is particularly important during NRP, as it allows for real-time assessment of whether the organs are receiving sufficient oxygen to meet their metabolic needs. Inadequate oxygenation, as reflected by a disproportionate increase in VCO_2_, can be an early indicator of metabolic distress, allowing for timely interventions to adjust perfusion parameters. The integration of DO_2*i*_ monitoring with O_2_ERi and VCO_2_ during NRP offers several clinical advantages that can significantly improve the outcomes of organ transplantation from DCD donors:

1. *Optimized Organ Preservation*: By ensuring that DO_2*i*_ is adequately matched to the metabolic demands of the organs, as reflected by O_2_ERi and VCO_2,_ clinicians can reduce the risk of ischemic injury and improve the overall quality of the organs being preserved for transplantation.
2. *Early Detection of Metabolic Imbalances*: Monitoring VCO_2_ in conjunction with DO_2*i*_ provides an early warning system for metabolic imbalances, such as hyperlactatemia, which can indicate insufficient oxygen delivery. This allows for timely adjustments to perfusion settings, potentially preventing irreversible organ damage.
3. *Personalized Perfusion Strategies*: The ability to monitor and adjust DO_2*i*_ in relation to O_2_ ERi and VCO_2_ enables the development of personalized perfusion strategies tailored to the specific needs of each donor. This approach can lead to more consistent and reliable outcomes in organ preservation and transplantation.
4. *Reduced Risk of Post-Transplant Complications*: By maintaining an optimal balance between oxygen delivery and metabolic demand, the incidence of post-transplant complications, such as AKI and delayed graft function, can be reduced, leading to better long-term outcomes for transplant recipients.

Although current monitoring programs and devices from companies like Terumo, Eurosets, LivaNova, and Spectrum Medical are primarily designed for application during cardiopulmonary bypass in cardiac surgery, the scenario of NRP in DCD donors presents a promising new frontier. This emerging field could open significant opportunities for these companies to adapt and expand their technologies, potentially developing specialized solutions that target the unique challenges of NRP, ultimately enhancing organ preservation and transplantation outcomes. The potential benefits of monitoring DO_2*i*_ in relation to O_2_ERi and VCO_2_ during NRP in DCD donors are substantial. Raising awareness about the importance of indexed DO_2_ calculations, especially in a clinical context where traditional tools like Swan-Ganz catheters are used less frequently due to the widespread adoption of echocardiography, could stimulate a more holistic approach to indexed calculations in perfusion management [4]. This shift in focus may encourage clinicians to re-emphasize the value of these calculations, integrating them more fully into practice, and potentially driving innovation in monitoring programs and devices tailored to advanced scenarios like NRP in DCD donors. These parameters provide a comprehensive view of the metabolic and oxygenation status of the organs, enabling clinicians to optimize perfusion strategies and improve the quality and viability of the organs being preserved for transplantation. As research in this area continues to evolve, the incorporation of these advanced monitoring techniques into routine clinical practice could significantly enhance the success rates of organ transplantation from DCD donors, ultimately expanding the donor pool and saving more lives.

## References

[1] de Somer F, Mulholland JW, Bryan MR, et al. O_2_ delivery and CO_2_ production during cardiopulmonary bypass as determinants of acute kidney injury: time for a goal-directed perfusion management? Crit Care. 2011;15(4):R192. 21831302 10.1186/cc10349 PMC3387634

[2] Ranucci M, Di Dedda U, Cotza M, Zamalloa Moreano K. The multifactorial dynamic perfusion index: A predictive tool of cardiac surgery associated acute kidney injury. Perfusion. 2024;39(1):201–209. 36305847 10.1177/02676591221137033 PMC10748450

[3] Condello I, Santarpino G, Nasso G, et al. Associations between oxygen delivery and cardiac index with hyperlactatemia during cardiopulmonary bypass. JTCVS Tech. 2020;2:92–99. 34317766 10.1016/j.xjtc.2020.04.001 PMC8299069

[4] Sanfilippo F, Noto A, Ajello V, et al. The use of pulmonary artery catheters and echocardiography in the cardiac surgery setting: a nationwide Italian Survey. J Cardiothorac Vasc Anesth. 2024;38(9):1941–1950. 38897888 10.1053/j.jvca.2024.04.046

